# Effective ascorbate-free and photolatent click reactions in water using a photoreducible copper(II)-ethylenediamine precatalyst

**DOI:** 10.3762/bjoc.11.211

**Published:** 2015-10-21

**Authors:** Redouane Beniazza, Natalia Bayo, Florian Molton, Carole Duboc, Stéphane Massip, Nathan McClenaghan, Dominique Lastécouères, Jean-Marc Vincent

**Affiliations:** 1 Université de Bordeaux, Institut des Sciences Moléculaires, UMR-CNRS 5255, 351 Crs de la Libération, 33405 Talence, France; 2 Univ. Grenoble Alpes, DCM UMR-CNRS 5250, F-38000 Grenoble, France; 3 Univ. Bordeaux, IECB, UMS 3033/US 001, 2 rue Escarpit, 33607 Pessac, France; 4 CNRS, IECB, UMS 3033, 33600 Pessac, France; 5 INSERM, IECB, US 001, 33600 Pessac, France

**Keywords:** benzophenone photosensitizer, bioconjugation, click chemistry, copper, photoreduction

## Abstract

The search for copper catalysts able to perform effectively click reactions in water in the absence of sodium ascorbate is an active area of current research with strong potential for applications in bioconjugation. The water-soluble and photoreducible copper(II)–EDA (EDA = ethylenediamine) complex **1**, which has two 4-benzoylbenzoates acting as both counterion and photosensitizer, has been synthesized and characterized by different techniques including single crystal X-ray diffraction. Highly efficient photoreduction was demonstrated when solutions of **1** in hydrogen atom donating solvents, such as THF or MeOH, were exposed to UVA radiation (350–400 nm) provided by a low pressure mercury lamp (type TLC = thin-layer chromatography, 365 nm), or by a 23 W fluorescent bulb, or by ambient/sunlight. In water, a much poorer hydrogen atom donating solvent, the photoreduction of **1** proved inefficient. Interestingly, EPR studies revealed that complex **1** could nonetheless be effectively photoreduced in water when alkynes were present in solution. The catalytic activity of **1** for click reactions involving a range of water-soluble alkynes and azides, in particular saccharides, was tested under various illumination conditions. Complex **1** was found to exhibit a photolatent character, the photogenerated copper(I) being very reactive. On irradiating aqueous reaction mixtures containing 1 mol % of **1** at 365 nm (TLC lamp) for 1 h, click reactions were shown to proceed to full conversion.

## Introduction

Since the discovery in 2002 that copper(I) could catalyze the Huisgen alkyne–azide [3 + 2] cycloaddition with high selectivity for the 1,4-triazole [1–2], the so-called copper(I)-catalyzed alkyne–azide cycloaddition (CuAAC) has become a privileged reaction which is widely employed in all areas of the chemical/biological/material sciences [3–4]. Numerous copper-based catalytic systems have been developed and employed for the CuAAC [5], the main prerequisite being the generation of a copper(I) catalytic species from various homogeneous/heterogeneous precatalysts, whose oxidation states are 0, +1 or +2. A major application of the CuAAC concerns bioconjugation reactions, i.e., the covalent modification of biomolecules [6]. Such reactions typically imply water-soluble alkyne and azide reactants and should thus be performed in an aqueous medium using a water-soluble catalyst. Important limitations for such transformations are: (i) high copper loading, often used in excess with respect to the substrates, due to limited catalyst reactivity and the fact that the substrates (proteins, oligonucleotides or oligosaccharides) are typically used in dilute conditions; (ii) contamination of the products by copper salts, which should be avoided for in vivo applications and, when employed, by sodium ascorbate and/or its byproducts; (iii) side-reactions on the substrates due to the generation of reduced dioxygen-active species and/or reactive oxidized byproducts of ascorbate. In a seminal paper, Finn and coworkers addressed several of these points; they proposed an optimized catalytic system composed of CuSO_4_, an accelerating and water-soluble tris-triazole THPTA (tris[(1-hydroxypropyl-1*H*-1,2,3-triazol-4-yl)methyl]amine) ligand with a ligand/copper ratio equal to at least 5 to effectively trap the reactive oxygenated species [7]. They also show that aminoguanidine could be added to the reaction mixture to effectively trap the reactive byproducts derived from ascorbate oxidation. Using these protective additives (excess of ligand and guanidine) bioconjugation reactions could be conducted from a Cu(II) precatalyst even when the reaction mixture is exposed to air. Optimized ligands leading to faster kinetics were later developed [8–9], allowing for instance to lower the copper loading, which is important to avoid toxicity issues for applications with living cells [9]. An interesting catalyst was reported by Gautier and coworkers based on a water-soluble Cu(I)–NHC complex, which could be used under ascorbate-free and open air conditions for the CuAAC ligation of oxidation-sensitive peptides in buffered aqueous media [10].

**Scheme 1:**
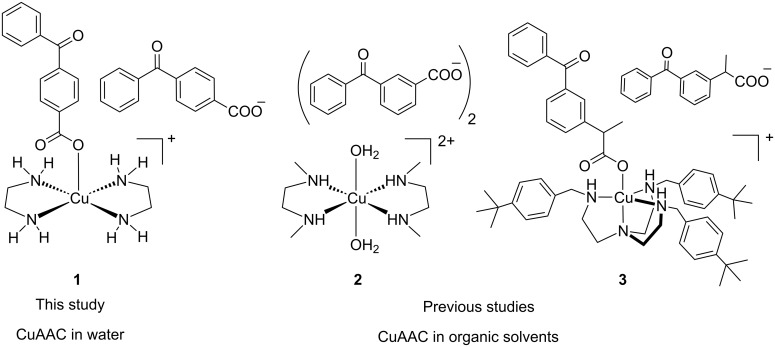
Structures of photoactivable click catalysts **1**–**3**.

Recently, we developed the photoreducible copper(II) complexes **2** and **3** incorporating a tren (tren = tris(2-aminoethyl)amine) ligand derivative [11–12] or the dmeda (dmeda = *N*,*N*’-dimethylethylenediamine) ligand [13–14] (Scheme 1). Irradiation at 365 nm of the benzophenone photosensitizer (n→π* electronic transition), introduced through the carboxylate counterion, mediated a highly efficient photoinduced electron transfer process leading to a fast Cu(II) to Cu(I) reduction, the final electron source being the solvent. The photoreduction process was extremely efficient, photoreduction quantum yields (Ф_red_) ranging from 0.17 up to around 1 being measured in good H-atom donating solvents such as MeOH or THF [11–13]. Consequently, efficient reduction could be achieved by simply exposing the solutions of the Cu(II) complexes to ambient light. Importantly, the photogenerated Cu(I) species were shown to be extremely reactive for the CuAAC reaction when conducting the reactions in organic solvents, typically MeOH, THF or toluene. It should be noted that within the last four years, other photoreducible copper(II)-based catalytic systems applied to click chemistry have been reported [15–27], in particular for the preparation of polymers [15–24].

We wish now to report on our preliminary studies dealing with the water-soluble complex **1**, the objective being to develop photoactivable click copper(II) precatalysts which could be used in aqueous solution and ascorbate-free conditions. We describe here the synthesis and characterization of **1**, its photoreduction properties in various solvents and illumination conditions, and its catalytic properties which reveal that **1**, when illuminated, is an effective photolatent click catalyst in aqueous medium.

## Results and Discussion

**Scheme 2:**
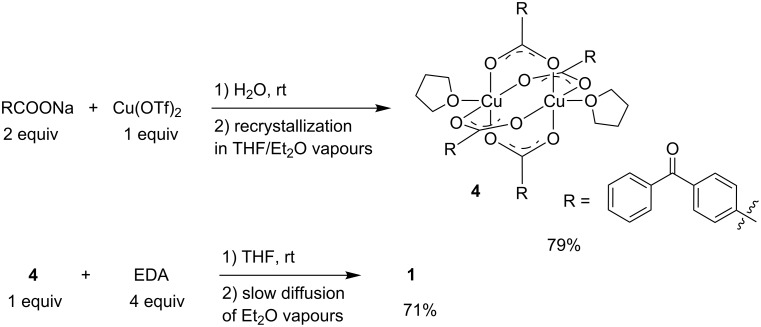
Syntheses of complexes **1** and **4**.

The EDA (EDA = ethylenediamine) ligand in the copper(II) complex [Cu^II^(EDA)_2_(4-benzoylbenzoate)](4-benzoylbenzoate) (**1**) was chosen to ensure high water solubility. Complex **1** was synthesized in two steps by first preparing the copper(II)-carboxylate dimer [Cu_2_(4-benzoylbenzoate)_4_(THF)_2_] **4** which was obtained by reacting 2 equivalents of the sodium salt of the 4-benzoylbenzoic acid with Cu(OTf)_2_ in water, the precipitate which formed being recrystallized by slow diffusion of Et_2_O in a THF solution (Scheme 2). Then, 4 equivalents of EDA were reacted with **4** in THF, the solution immediately turned deep blue. Slow diffusion of diethyl ether vapour into the THF solution led to the crystallization of **1** as blue needles, which were recovered by filtration (71% yield).

**Figure 1:**
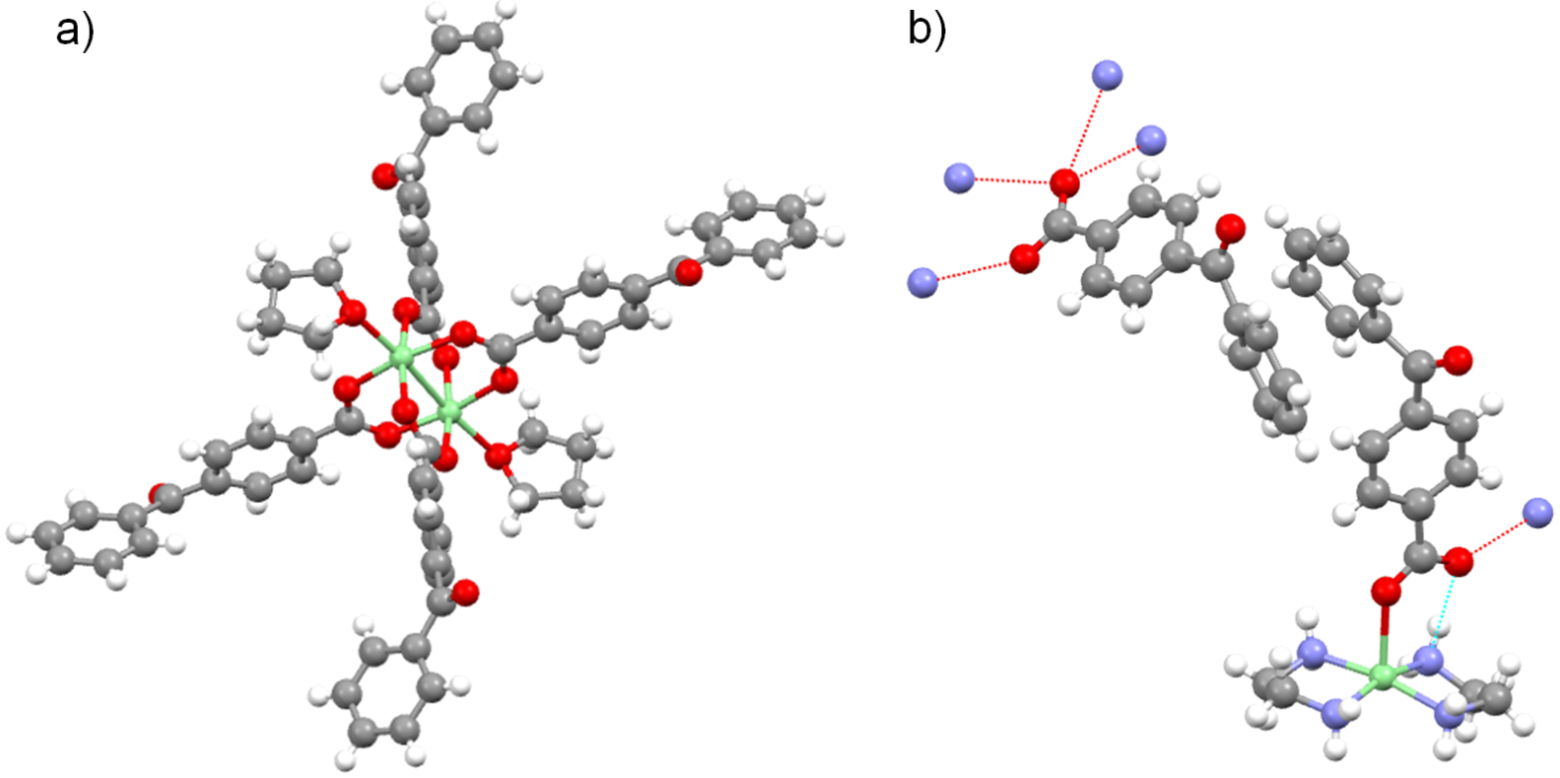
a) Molecular structure of **4** (a THF molecule present in the unit cell is not shown). Cu, green; C, grey; O, red; H, white; b) Molecular structure of **1** (asymmetric unit) showing the intermolecular (dashed red lines) and intramolecular (dashed blue line) H-bond interactions of the carboxylate C=O bonds with surrounding H–N bonds. Cu, green; N, blue; C, grey; O, red; H, white. CIF files for the determined structures are available as Supporting Information File 1 and Supporting Information File 2 and are also available on request from the Cambridge Crystallographic Data Centre as deposition CCDC 1414149 for **4**, and CCDC 1410011 for **1**.

The proposed dinuclear and mononuclear structures of **4** and **1** were confirmed by single crystal X-ray diffraction (Figure 1). The crystal structure of **4** displays the typical “paddle-wheel” of copper(II)–carboxylate complexes [28], a structure in which the two copper(II) ions are bridged by four carboxylates in a *syn–syn* configuration (*d*_Cu···Cu_ 2.613 Å), whilst two THF molecules occupy axial coordination sites.

Complex **1** displays a monomeric structure with a distorted square pyramidal geometry with the copper ion lying slightly above the basal plane formed by 4 nitrogen atoms (average *d*_Cu···N_ 2.019 Å), while an oxygen atom of a carboxylate occupies the axial site (*d*_Cu···O_ 2.294 Å). The oxygen atom of the C=O bond is participating in one intramolecular (*d*_CO···HN_ 2.140 Å) and one intermolecular (*d*_CO···HN_ 2.212 Å) hydrogen bond with hydrogen atoms of EDA ligands. The second carboxylate anion is not directly bound to the copper(II) ion, but participates in four intermolecular hydrogen bonds with hydrogen atoms of EDA (average *d*_CO···HN_ 2.125 Å) ligands of two [Cu(EDA)_2_(4-benzoylbenzoate)]^+^ cations.

**Figure 2:**
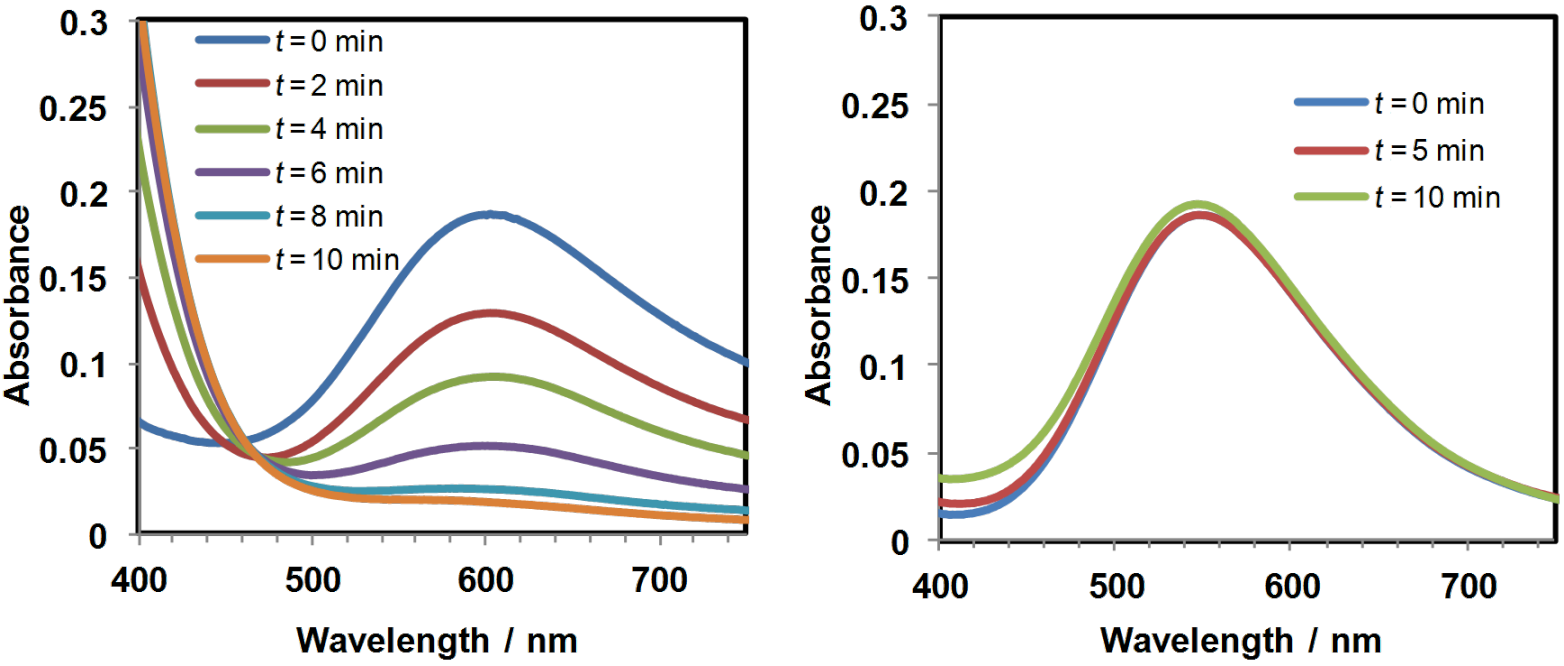
Evolution of the UV–vis spectra of deaerated (freeze-pump-thaw degassed, sealed quartz cuvettes) THF (left) or water (right) solutions (3 mL) of complex **1** (2 mM) under irradiation at 365 nm using a TLC lamp placed at ≈1 cm from the cuvette.

Aqueous and THF solutions of **1** were characterized by a shift of the absorption band (d–d electronic transition) in the visible spectral region from 550 nm in water to 606 nm in THF (spectra at *t* = 0 min in Figure 2). This was ascribed to the ion-pair dissociation state of the complex with water molecules being most probably bound to the copper ions in aqueous solution, while in THF, the carboxylates interact more tightly with the copper ions. This is in agreement with the ^1^H NMR spectra of **1**, which showed well resolved peaks for the benzophenone protons in D_2_O, while in THF-*d*_8_ broad resonances were observed, as expected for a compound interacting more strongly with paramagnetic copper(II) ions. It should be noted that the methylene protons of the EDA ligands are not observed.

Photoreduction studies of **1** were then conducted varying both the solvent and illumination conditions. The first experiments were carried out in quartz cuvettes to follow the disappearance of the low-energy visible light absorption band (550–600 nm) ascribed to a d–d electronic transition, which is typical for Cu^II^ complexes (d^9^). The reductions were typically conducted under strictly anaerobic conditions, deaeration of the solutions being achieved either by gentle Ar bubbling through a rubber cap or, by freeze-pump-thaw cycles followed by sealing of the cell. Irradiations were performed at wavelengths compatible with the n→π* electronic transition of the benzophenone chromophore, i.e., ≈350–370 nm (UVA). Illumination of the samples at 365 nm using a TLC lamp thus represents a convenient source of light. Previous studies revealed that a 2 mM solution of **2** in THF (3 mL in a quartz cuvette with 1 cm path length) was fully reduced in ≈15 min [14]. Interestingly, the photoreduction process was found to be so effective, that the UVA photons (350–400 nm) present in sunlight/ambient light were sufficient to achieve the reduction with satisfactory rates, i.e., ≈30 min and 60 min when the samples were exposed behind a window to direct light of a sunny or a rainy day, respectively. It should also be noted that over-reduction processes leading to the formation of copper(0) nanoparticles occurred when prolonged irradiation times were applied [12–13].

When a deaerated solution of **1** in THF was irradiated at 365 nm, a very fast change of the solution aspect was observed, the solution becoming colourless in ≈10 min as shown by UV–vis spectroscopy (Figure 2). This rate is comparable to that observed previously with the analogous complex **2**, for which a photoreduction quantum yield close to unity has been determined in THF [14]. Because of such a high efficiency, the photoreduction proceeded well under direct sunlight illumination by placing the quartz cuvette behind a window of a sunny day, ≈60% of **1** being reduced in 40 min illumination under such conditions.

When irradiations were conducted on aqueous solutions of **1**, no reduction was observed, as revealed in Figure 2. This agrees with the poor hydrogen atom donating character of H_2_O (BDE = 119 kcal/mol) compared to THF (BDE = 92 kcal/mol), the reactivity of the excited triplet of benzophenone being particularly high toward the THF moiety [29].

**Scheme 3:**
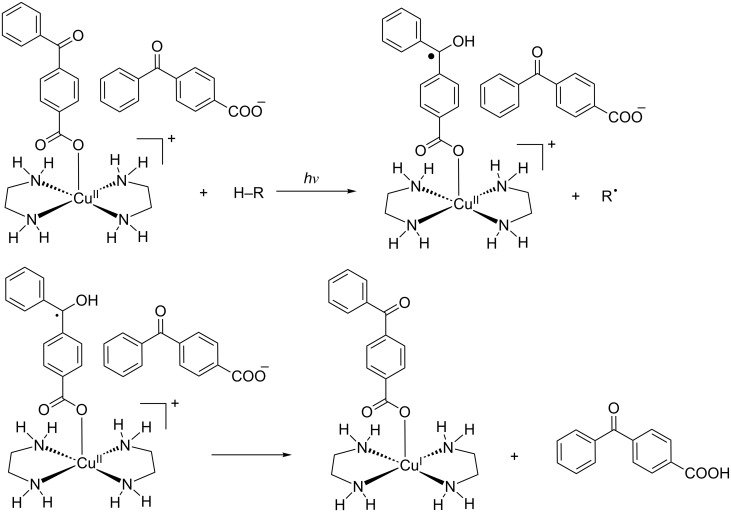
Proposed mechanism for the photoreduction process.

At this stage, a reduction mechanism implying the fast generation of the ketyl radical in good hydrogen atom donating solvents, which then can reduce the copper(II) ion to generate the copper(I) and regenerate the benzophenone chromophore, could be proposed (Scheme 3). Further studies, which aim to validate this proposal, are in progress.

The poor reduction efficiency observed in water could represent a serious limitation for our goal to develop photolatent click catalysts for reactions conducted in water with water-soluble reactants. However, under catalytic conditions, i.e., in the presence of a large excess of alkyne and azide (1 mol % of **1** will be used for catalytic reactions), the reactants could be used as reducing agent. For instance, it has been previously shown that the alkynes could favor the Cu(II) to Cu(I) reduction, most probably through the well-known Glaser-type oxidative coupling [30–31]. We thus tested the reduction in the presence of the water-soluble alkynes **5**–**7**. In marked contrast with experiments conducted in pure water, the aqueous solution irradiated in the presence of alkyne **5** (50 equiv with respect to copper), rapidly evolved to become slightly cloudy. Such changes, which could be ascribed to the formation of insoluble polymeric copper(I) acetylides and/or the insoluble 3-benzoylbenzoic acid (Scheme 3), precluded the use of UV–vis spectroscopy to quantitatively analyze the reductive process. For such an analysis, EPR spectroscopy was thus employed to assess the extent of copper reduction.

**Figure 3:**
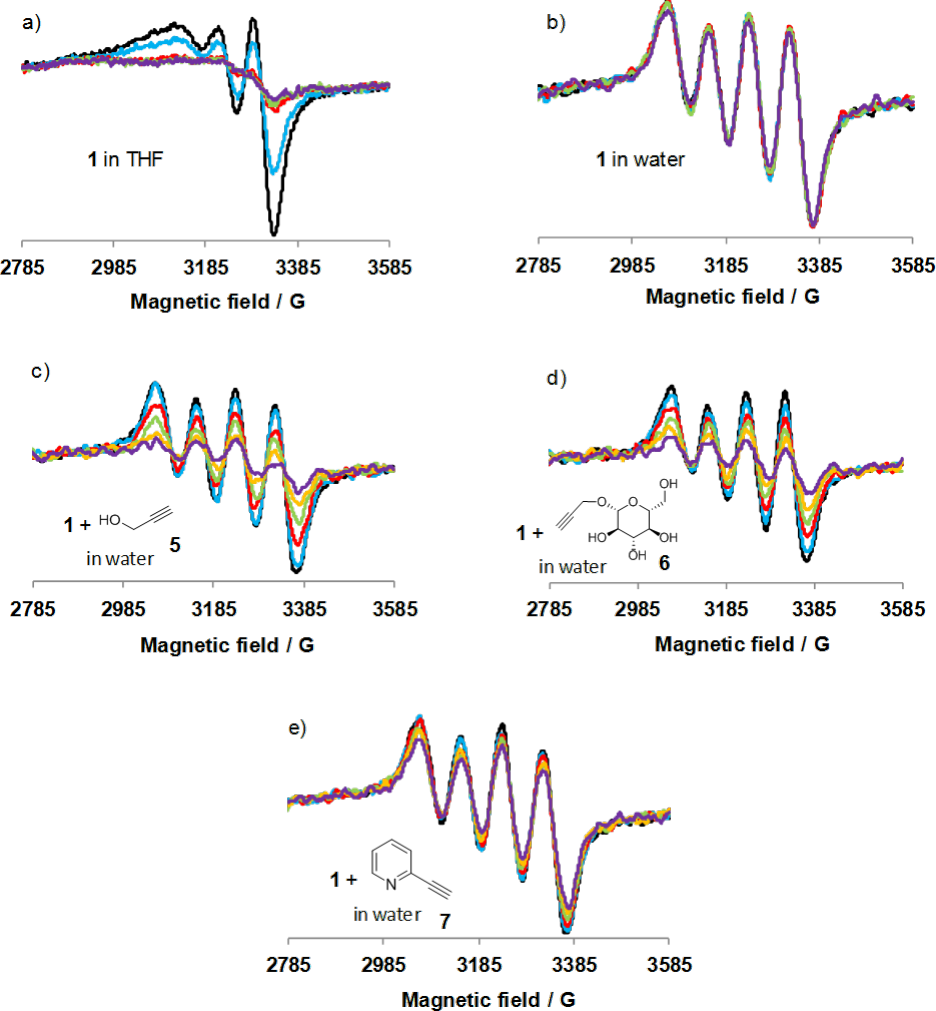
Evolution of the EPR spectra (X band, 298 K) of solutions of **1** under continuous irradiation (280–400 nm). A spectrum is recorded in the dark (black), then the light is switched on and the spectra (scanning time = 15 s) recorded during irradiation at times 0 s (blue), 50 s (red), 100 s (green), 150 s (orange) and 225 s (purple). a) THF solution of **1** (1 mM); b) H_2_O solution of **1** (1 mM); c) H_2_O solution of **1** (1 mM) and alkyne **5** (50 mM); d) H_2_O solution of **1** (1 mM) and alkyne **6** (50 mM); e) H_2_O solution of **1** (1 mM) and alkyne **7** (50 mM).

In Figure 3, the EPR spectra of solutions of **1** (d^9^, *S* = 1/2) recorded by irradiating the samples directly in the probe at room temperature are presented. Control experiments conducted in THF and aqueous solutions of **1** (Figure 3a and b, respectively), confirmed the results gathered by UV–vis spectroscopy, i.e., fast reduction in THF and essentially no reduction in water. In water solution, the EPR spectra displays the four well-resolved hyperfine lines, expected for Cu(II) characterized by a nuclear spin of 3/2. However, the shape of the EPR spectra are different in both solvents in agreement with the UV–vis data, i.e., a water molecule should replace the Cu-bound benzoylbenzoate in aqueous solution.

When the water-soluble alkynes **5**, **6** or **7** were added (50 equiv with respect to Cu) and the aqueous solution irradiated, a decrease of the intensity of the EPR signal was observed, in agreement with a reduction process (Figure 3c–e). In the presence of propargyl alcohol **5** or propargyl ether **6** the reductions occurred very efficiently, i.e., ≈65% of the copper(II) (estimated by integration of the EPR signal) was reduced after 250 s of irradiation. This is nonetheless slower than in THF, in which ≈65% of **1** was reduced after only 40 s. With 2-alkynylpyridine **7**, reduction was also observed albeit at a slower rate, ≈35% of **1** being reduced after 250 s of irradiation. It is important to note that no reduction was observed in the absence of light, showing that the reduction is a photoinduced process. It also shows that if light-independent reduction is occurring, most probably through a Glaser-type oxidative coupling, it is a much slower process. At this stage, we propose that the alkynes serve as the hydrogen atom source to generate the ketyl radical of the benzophenone (Figure 2).

**Figure 4:**
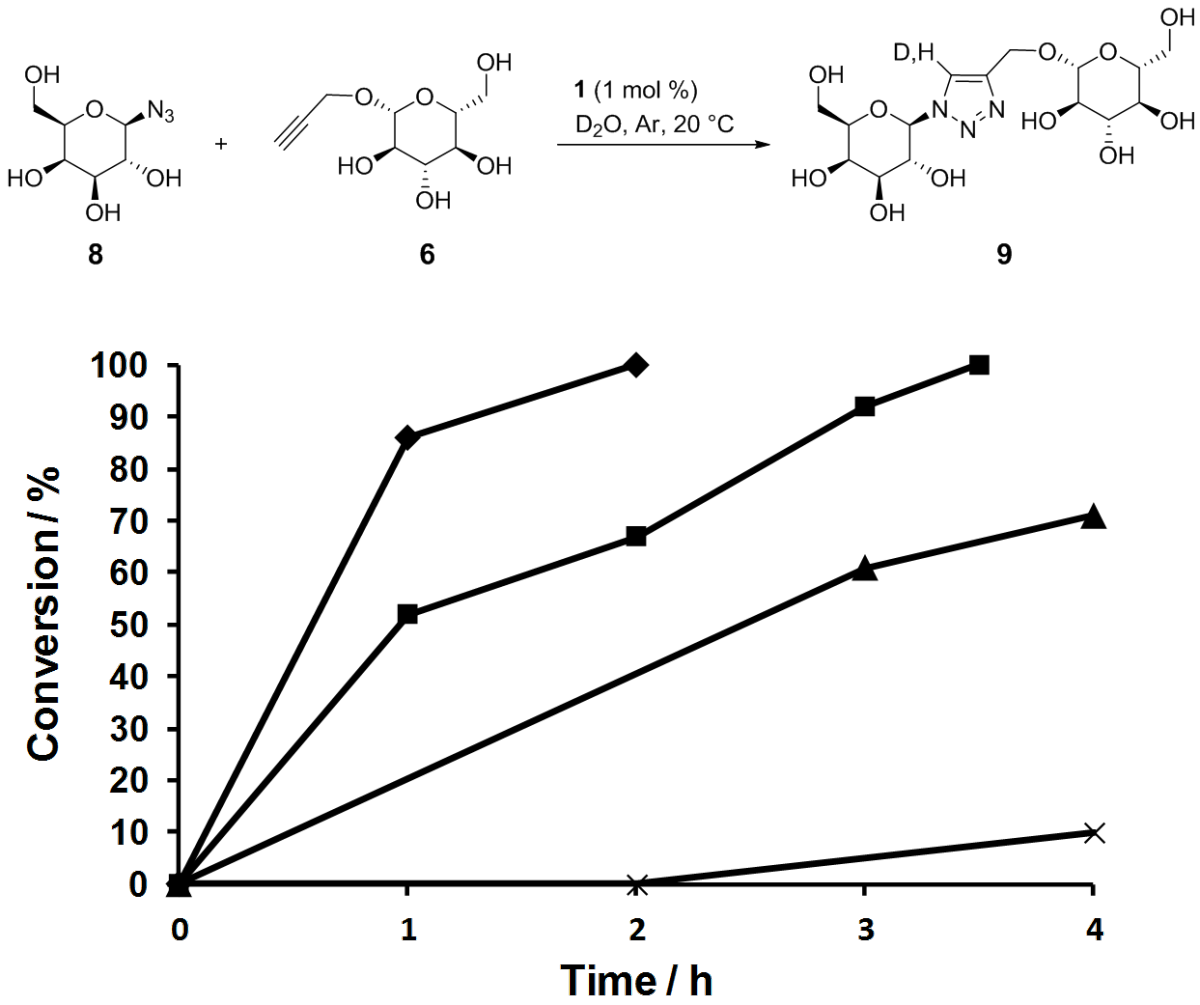
Reaction profiles for the formation of **9** under various illumination conditions: TLC lamp (365 nm) for 1 h, then ambient laboratory light (♦); tube in the dark (X); tube exposed to ambient laboratory light (▲); tube exposed to 23 W fluorescent bulb (■).

The photolatent properties of **1** and the reactivity of the photogenerated copper(I) species were then tested under various illumination conditions (Figure 4). Reactions between the water-soluble alkyne **6** and azide **8** were conducted in D_2_O in NMR tubes, thereby allowing convenient monitoring of the reaction progress. In a typical experiment, the NMR tube was charged with **1** (1 mol %), D_2_O (0.5 mL), the reactants (0.15 mmol each) and capped with a rubber septum. The whole tube was protected from light by aluminum foil, the solution was degassed by gentle Ar bubbling (20 min), and the tube was tightly capped with parafilm to limit air entry. The reactions were initiated by exposing the tubes to various light sources. In a first experiment the solution was irradiated for 1 h at 365 nm with a TLC lamp placed at ≈1 cm from the tube, and then left under ambient laboratory light. In these conditions, the reaction proceeded to full conversion in ≈2 h, 80–90% of the triazole being formed after 1 h. The triazole **9** was then isolated in 91% yield. Importantly and in marked contrast, when the tube was protected from light, no reaction occurred within 2 h, highlighting the photolatent behavior of **1**. However a slow reaction was observed, ≈10% conversion was obtained in 4 h, and 74% in 8 h. This could be ascribed to the light-independent slow generation of copper(I) through Glaser-type oxidative coupling.

The catalytic system was found to be very sensitive to light. By simply leaving the tube exposed to ambient laboratory light after degassing, 70% conversion was reached in 4 h, the full conversion being attained in 7 h. Interestingly, from a practical viewpoint, it is possible to simply illuminate the sample with a household fluorescent bulb (23 W) which produces a significant amount of UVA photons. Using this light source, full conversion was achieved in 3.5 h, which is faster than when exposed to ambient light, but significantly slower than when exposed to TLC lamp.

**Scheme 4:**
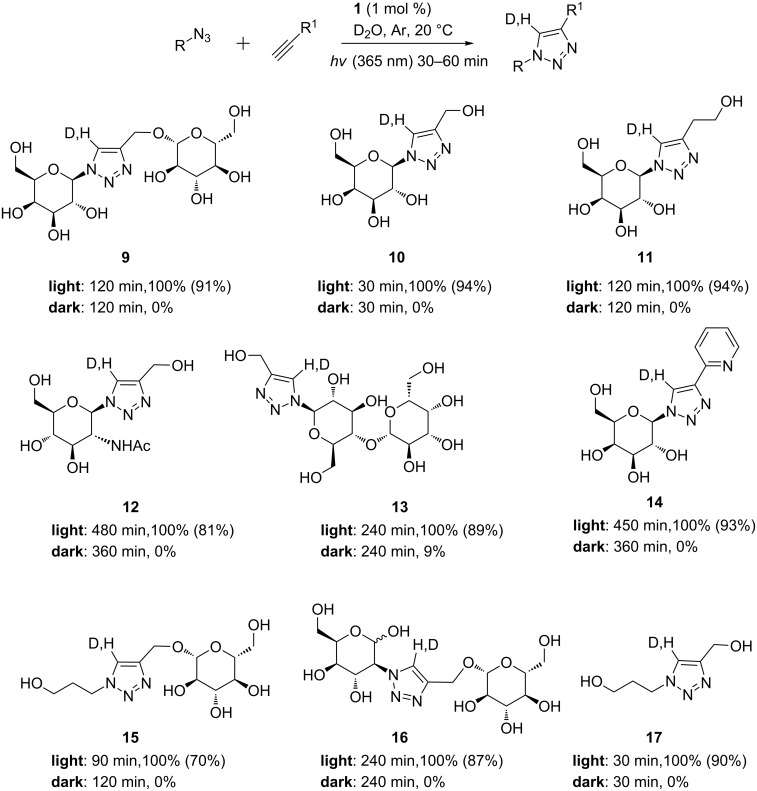
Structures, conversions and isolated yields for triazoles **9**–**17** conducted in D_2_O in NMR tubes.

Having established the most efficient illumination conditions, reactions were conducted on a range of water-soluble alkynes and azides by irradiating the NMR tubes with the TLC lamp for 1 h and then leaving it under ambient light. As seen in Scheme 4, a variety of triazoles **9**–**17** could be obtained in good isolated yields with reaction times ranging from 30 min to 8 h. Again, no or little reaction was observed when protecting the tube from light, in agreement with photolatent catalysis.

**Scheme 5:**
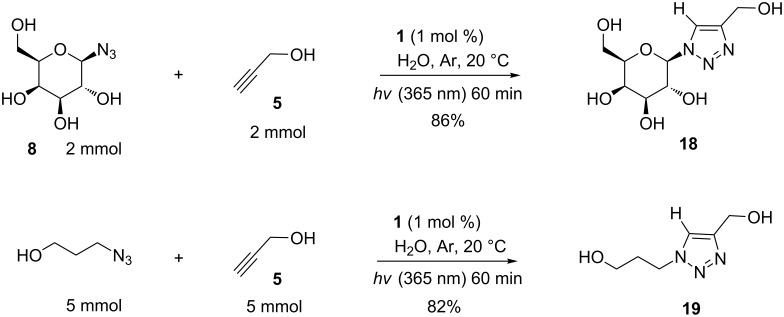
Preparative scale synthesis of **18** and **19**.

Finally, two reactions were conducted in H_2_O on preparative scales in round-bottom flasks (Scheme 5). The hydrogenated triazoles **18** and **19** were obtained in 86% (0.450 g) and 82% (0.644 g) isolated yields, respectively, showing that the procedure is practical for laboratory-scale applications.

## Conclusion

The copper(II) precatalyst **1** incorporating a benzophenone chromophore is easily prepared, soluble in polar solvents and water, and can be stored indefinitely under ambient conditions. It is efficiently photoreduced in THF using convenient light sources producing UVA photons. While the Cu(II) to Cu(I) reduction process in water proved inefficient we have shown, in particular using EPR spectroscopy, that the alkynes can serve as effective and practical electron source to allow the reduction in a light-dependent process photosensitized by the benzophenone. Complex **1** proved to be an effective photolatent catalyst for reactions conducted in water involving water-soluble alkynes and azides. Combining **1** with light, thus prevents the use of sodium ascorbate as reducing agent while a copper(II) complex is employed as precatalyst. The reactions were conducted not only under ascorbate-free conditions, but also under dioxygen-free conditions, deoxygenation being conveniently performed by Ar or N_2_ bubbling. Copper(I)-catalyzed processes, in particular for application in click bioconjugations, should be conducted under deaerated conditions in order to reduce copper loading due to fast Cu(I) to Cu(II) oxidation process mediated by O_2_, and also to limit addition of trapping agents to capture reactive oxygen species generated through side reactions between Cu(I) and O_2_.

## Supporting Information

File 1 Experimental and analytical data.

File 2 Crystallographic Information for compound **1**.

File 3 Crystallographic Information for compound **4**.
