# Supplementary material for: Effective ascorbate-free and photolatent click reactions in water using a photoreducible copper(II)-ethylenediamine precatalyst
Source: Beilstein J Org Chem. 2015 Oct 21;11:1950–9. doi: 10.3762/bjoc.11.211 (PMC4661010; doi:10.3762/bjoc.11.211)
Supplement: File 1 — Experimental and analytical data. [file Beilstein_J_Org_Chem-11-1950-s001.pdf]

Supporting Information

for

Effective ascorbate-free and photolatent click reactions in water using a photoreducible copper(II)-ethylenediamine precatalyst

Redouane Beniazza[1], Natalia Bayo[1], Florian Molton[2], Carole Duboc[2], Stéphane Massip[3,4,5], Nathan McClenaghan[1], Dominique Lastécouères[1] and Jean-Marc Vincent*[1]

Address:[1] Université de Bordeaux, Institut des Sciences Moléculaires, UMR-CNRS 5255, 351 Crs de la Libération, 33405 Talence, France, [2]Univ. Grenoble Alpes, DCM UMR-CNRS 5250, F-38000 Grenoble, France, [3]Univ. Bordeaux, IECB, UMS 3033/US 001, 2 rue Escarpit, 33607 Pessac, France, [4]CNRS, IECB, UMS 3033, 33600 Pessac, France and [5]INSERM, IECB, US 001, 33600 Pessac, France

Email: Jean-Marc Vincent* - jm.vincent@ism.u-bordeaux1.fr

* Corresponding author

Experimental and analytical data

General

All reagents were obtained from commercial sources and were used as received, except for the propargyl alcohol which was distilled prior to use. Solvents were dried and distilled prior to use. The alkyne **6** [1] and azide **8** [2] were prepared following published procedures. NMR analyses were carried out on Bruker avanceII-200 and Bruker avanceII-300 spectrometers in deuterated methanol or water as solvent. The chemical shifts (δ) for carbon and proton are given compared to the residual solvent peak and are expressed in ppm. Elemental analyses were performed at the analytical center of the Département de Chimie Moléculaire of the University Grenoble Alpes. Results are expressed in weight percent. Photoirradiation at 365 nm was performed with a portable Fisher Bioblock low pressure mercury lamp (type Thin Layer Chromatography “TLC”) with a power of 6 watts. Absorption spectra were recorded on a Varian Cary 5000 spectrophotometer in quartz cells with 1 cm path length. Degassing was achieved through multiple freeze-pump-thaw cycles, before blowtorch sealing the cells.

EPR measurements

The samples were kept in the dark all along the experiment before irradiation. In a glove-box, under inert atmosphere of argon, complex **1** (1 mM, 0.34 mg) was dissolved in 500 µL of degassed deionized water (or degassed THF). 50 equivalents of alkyne (1.5 µL of propargyl alcohol **5**, 5.1 mg of propargyl ether **6** or 2.5 µL of alkynylpyridine **7**) were added to the solution. 150 µL of each solution were transferred to a closed Pasteur pipet then tightly capped. The pipet was then inserted in the EMX Bruker spectrometer equipped with a X-band ER4102ST Bruker cavity for in situ measurement at 298 K. The frequency was 9.44 GHz and the power was 22 mW. Each scan lasted 15 s following by 10 s of delay. Continuous irradiation was applied after the end of the first scan using a 150 W Xenon lamp equipped with a 280–400 nm filtering mirror from Newport.

Synthesis of [Cu_2(4-benzoylbenzoate)$_4$(THF)$_2$] (4)

An aliquot (3.26 mL, 9.8 mmol) of a NaOH solution (3 M) was added to a suspension of 4-benzoylbenzoic acid (2.2 g, 9.8 mmol) in water (50 mL). The solution was stirred until it became homogeneous, then $Cu(CF_3SO_3)_2$ (1.77 g, 4.9 mmol) was added. After stirring for 30 min the resulting blue-green precipitate was filtered, washed with water and dried under vacuum affording a blue-green powder (2.6 g). This was dissolved in dry THF (200 mL), followed by slow diffusion of diethyl ether vapour which led to the formation of turquoise X-ray quality crystals of **4** (2.27g, 79%). ATR-FTIR (crystal): 2975 (br), 2868, 1668, 1627, 1596, 1579, 1562, 1502, 1446, 1411, 1314, 1274, 1175, 1153, 1139, 1037, 939, 926, 871, 845, 775, 722, 696. M.p.: 254-255 °C.

Synthesis of [Cu(EDA)$_2$(4-benzoylbenzoate)](4-benzoylbenzoate) (1)

On adding ethylenediamine (0.17 mL, 2.56 mmol) to a solution of [Cu_2(4-benzoylbenzoate)$_4$(THF)$_2$] **4** (750 mg, 1.28 mmol of Cu^{2+} ions) in THF (32 mL), the solution turns immediately to deep blue. After 30 min of stirring, slow diffusion of diethyl ether afforded **1** as blue needles (574 mg, 71%), which were recovered by filtration. Complex **1** was found to be hygroscopic, as revealed by elemental analysis and IR measurement showing a broad band from 3100 to 3650 cm^{-1} corresponding to O–H stretching vibrations. Elemental Analyses: Calcd. for $C_{32}H_{34}CuN_4O_6.4H_2O$: C, 54.42; H, 5.99; N, 7.93. Found (%): C, 53.90; H, 6.00; N, 7.79. ATR-FTIR (crystal): 3385 (br), 3324, 3266 (br), 3158, 2953, 2888, 1650, 1615, 1588, 1538, 1496, 1457, 1447, 1384, 1318, 1278, 1185, 1150, 1128, 1105, 1042, 1001, 983, 941, 929, 891, 878, 836, 812, 779, 715, 697. M.p.: 102-104 °C.

Procedure for the click reactions in NMR tubes

Complex **1** (1 mol %, 1.5 μmol, 0.95 mg) and the azide (0.15 mmol) were dissolved in D_2O (0.5 mL) in a vial. The resulting solution was then transferred to the NMR tube, the alkyne (0.15 mmol) was added and the tube capped with a rubber septum. The reaction mixture, protected from light by aluminum foil, was then degassed by gentle argon bubbling for 20

minutes, tightly capped using parafilm, and then irradiated at 365 nm for 30 or 60 min using a TLC lamp placed at ~1 cm from the tube. Once the reaction was complete, the deuterated solvent was evaporated and the residue was then taken up with MeOH. Adding ethyl acetate led to the precipitation of the triazoles which were recovered by filtration.

Preparative scale syntheses are described below for triazoles **18** and **19**.

(2*R*,3*S*,4*S*,5*R*,6*R*)-2-(Hydroxymethyl)-6-((1-((2*R*,3*R*,4*S*,5*R*,6*R*)-3,4,5-trihydroxy-6-(hydroxymethyl)tetrahydro-2*H*-pyran-2-yl)-1*D*-1,2,3-triazol-4-yl)methoxy)tetrahydro-2*H*-pyran-3,4,5-triol (9) [3]**:** Synthesized according to the general procedure from a mixture of azide **7** (31 mg, 0.15 mmol) and alkyne **6** (33 mg, 0.15 mmol) in D_2O (0.5 mL) for 120 min at 20 °C. The desired triazole **9** was obtained as a white powder (58 mg, 91%).

M.p. 77-78 °C; ^{1}H-NMR (D_2O, 200 MHz) δ (ppm) = 8.34 (s, 0.20 H), 5.60 (d, *J* = 9.2 Hz, 1H), 4.86 (dd, *J* = 12.8 Hz and *J* = 24.0 Hz, 2H), 4.46 (d, *J* = 7.8 Hz, 1H), 4.13 (d, *J* = 9.6 Hz, 1H), 3.98 (d, *J* = 3.2 Hz, 1H), 3.90 (t, *J* = 6.2 Hz, 1H), 3.83-3.73 (m, 2H), 3.72-3.70 (m, 3H), 3.40-3.08 (m, 4H); ^{13}C-NMR (D_2O, 50 MHz) δ (ppm) = 143.8, 123.8, 101.5, 88.0, 78.3, 75.9, 75.7, 73.0, 72.9, 69.7, 69.5, 68.5, 61,9, 60.8, 60.7; HRMS (ESI): Calcd. for $C_{15}H_{24}DN_3O_{11}$ $[M+H]^+$: 424.1551; Found: 424.1561.

(2*R*,3*R*,4*S*,5*R*,6*R*)-2-(4-(2-Hydroxyethyl)-1*D*-1,2,3-triazol-1-yl)-6-(hydroxymethyl)tetrahydro-2*H*-pyran-3,4,5-triol (11): Synthesized according to the general procedure from a mixture of azide **8** (31 mg, 0.15 mmol) and but-3-yn-1-ol (11.34 µL, 0.15 mmol) in D_2O (0.5 mL) for 120 min. at 20 °C. The desired triazole **11** was obtained as a brownish oil (36.3 mg, 94%).

^{1}H-NMR (D_2O, 200 MHz) δ (ppm) = 7.96 (s, 0.21 H), 5.52 (d, *J* = 8.8 Hz, 1H), 4.07 (t, *J* = 9.0 Hz, 1H), 3.95 (d, *J* = 3.0 Hz, 1H), 3.86 (t, *J* = 6.0 Hz, 1H), 3.77-3.59 (m, 5H), 2.83 (t, *J* = 6.2 Hz, 2H); ^{13}C-NMR (D_2O, 50 MHz) δ (ppm) = 145.7, 122.7, 87.9, 78.2, 72.8, 69.7, 68.5, 60.8, 60.4, 27.7; HRMS (ESI): Calcd. for $C_{10}H_{15}DN_3O_6Na$ $[M+Na]^+$: 298.0999; Found: 298.1012.

***N*-((2*R*,3*R*,4*R*,5*S*,6*R*)-4,5-Dihydroxy-6-(hydroxymethyl)-2-(4-(hydroxymethyl)-1*D*-1,2,3-triazol-1-yl)tetrahydro-2*H*-pyran-3-yl)acetamide (12):** Synthesized according to the general procedure from a mixture of azide (31 mg, 0.15 mmol) and propargyl alcohol **5** (8.7 µL, 0.15 mmol) in D_2O (0.5 mL) for 480 min. at 20 °C. The desired triazole **12** was obtained as a white powder (36.5 mg, 81%).

M.p. 157-159 °C; ^{1}H -NMR (CD_3OD, 300 MHz) δ (ppm) = 8.14 (s, 0.10H), 5.81 (d, *J* = 9.9 Hz, 1H), 4.69 (s, 2H), 4.25 (t, *J* = 9.9 Hz, 1H), 3.96-3.85 (m, 1H), 3.83-3.69 (m, 2H), 3.67-3.54 (m, 2H), 1.81 (s, 3H); ^{13}C-NMR (CD_3OD, 75.5 MHz) δ (ppm) = 172.6, 148.5, 122.4, 86.7, 79.5, 74.1, 69.7, 60.7, 55.3, 54.9, 21.3; HRMS (ESI): Calcd. for $C_{11}H_{17}DN_4O_6$ $[M+H]^+$: 303.1289; Found: 303.1291.

(2*S*,3*R*,4*S*,5*R*,6*R*)-2-((2*R*,3*S*,4*R*,5*R*,6*R*)-4,5-Dihydroxy-2-(hydroxymethyl)-6-(4-(hydroxymethyl)-1*D*-1,2,3-triazol-1-yl)tetrahydro-2*H*-pyran-3-yloxy)-6-(hydroxymethyl)tetrahydro-2*H*-pyran-3,4,5-triol (13) [4]**:** Synthesized according to the general procedure from a mixture of azide (55 mg, 0.15 mmol) and propargyl alcohol (**5**, 8.7 µL, 0.15 mmol) in D_2O (0.5 mL) for 240 min at 20 °C. The desired triazole **13** was obtained as a yellow powder (56.5 mg, 89%).

M.p. 91-92 °C; ^{1}H -NMR (D_2O, 300 MHz) δ (ppm) = 8.19 (s, 0.23H), 5.76 (d, *J* = 9.0 Hz, 1H), 4.71 (s, 2H), 4.47 (d, *J* = 7.8 Hz, 1H), 4.11-4.01 (m, 1H), 3.96-3.70 (m, 9H), 3.66 (dd, *J* = 9.9 Hz and *J* = 3.3 Hz, 1H), 3.55 (dd, *J* = 9.6 Hz and *J* = 7.5 Hz, 1H); ^{13}C-NMR (D_2O, 75.5 MHz) δ (ppm) = 149.4, 125.6, 106.4, 89.7, 80.2, 77.9, 77.0, 75.0, 74.4, 73.5, 71.1, 63.6, 62.2, 57.1; HRMS (ESI): Calcd. for $C_{15}H_{24}DN_3O_{11}Na$ $[M+H]^+$: 424.1551; Found: 424.1551.

2*R*,3*R*,4*S*,5*R*,6*R*)-2-(Hydroxymethyl)-6-(4-(pyridin-2-yl)-1*D*-1,2,3-triazol-1-yl)tetrahydro-2*H*-pyran-3,4,5-triol (14) [5]**:** Synthesized according to the general procedure from a mixture of azide (31 mg, 0.15 mmol) and 2-ethynylpyridine **7** (15.13 µL, 0.15 mmol) in D_2O (0.5 mL)

for 450 min. at 20 °C. The desired triazole **14** was obtained as a brownish powder (43 mg, 93%).

M.p. 77-79 °C; ^{1}H -NMR (CD_3OD, 200 MHz) δ (ppm) = 8.29 (s, 0.20H), 8.23-7.93 (m, 1H), 7.61-7.36 (m, 2H), 7.14-6.93 (m, 1H), 5.56 (d, *J* = 9.0 Hz, 1H), 4.13 (t, *J* = 9.4 Hz, 1H), 3.96 (d, *J* = 2.8 Hz, 1H), 3.89 (t, *J* = 5.8 Hz, 1H), 3.79-3.64 (m, 3H); ^{13}C-NMR (CD_3OD, 75.5 MHz) δ (ppm) = 149.5, 149.1, 147.4, 147.2, 137.5, 123.1, 120.3, 89.0, 78.7, 73.9, 70.0, 69.0, 61.3; HRMS (ESI): Calcd. for $C_{13}H_{15}DN_4O5$ $[M+H]^+$: 309.1183; Found: 309.1181.

(2*R*,3*S*,4*S*,5*R*,6*R*)-2-(Hydroxymethyl)-6-((1-(3-hydroxypropyl)-1*D*-1,2,3-triazol-4-yl)methoxy)tetrahydro-2*H*-pyran-3,4,5-triol (15):

Synthesized according to the general procedure from a mixture of 3-azidopropan-1-ol (30.3 mg, 0.3 mmol) and alkyne (65.4 mg, 0.33 mmol) in D_2O (1 mL) for 90 min. at 20 °C. The desired triazole **15** was obtained as a yellow powder (67 mg, 70%).

M.p. 146-148 °C; ^{1}H-NMR (D_2O, 200 MHz) δ (ppm) = 7.96 (s, 0.10 H), 4.91-4.71 (m, 2H), 4.56-4.32 (m, 3H), 3.86-3.72 (m, 1H), 3.60 (dd, *J* = 12.8 Hz and *J* = 5.2 Hz, 1H), 3.46 (d, *J* = 6.2 Hz, 2H), 3.39-3.10 (m, 4H), 2.02 (t, *J* = 8.6 Hz, 1H); ^{13}C-NMR (D_2O, 50 MHz) δ (ppm) = 150.6, 123.8, 101.4, 75.9, 75.7, 73.0, 69.6, 61,9, 60.7, 58.1, 47.2, 31.7. HRMS (ESI): Calcd. for C12H21DN3O7 [M+H]+:321.1515; Found: 321.1521.

(3*S*,4*S*,5*R*,6*R*)-6-(Hydroxymethyl)-3-(4-(((2*R*,3*R*,4*S*,5*S*,6*R*)-3,4,5-trihydroxy-6-(hydroxymethyl)tetrahydro-2*H*-pyran-2-yloxy)methyl)-1*D*-1,2,3-triazol-1-yl)tetrahydro-2*H*-pyran-2,4,5-triol (16): Synthesized according to the general procedure from a mixture of azide (31 mg, 0.15 mmol) and alkyne **6** (33 mg, 0.15 mmol) in D_2O (0.5 mL) for 4 h at 20 °C. The triazole **16** was obtained as a yellow foam (55 mg, 87%), and as a mixture of two diastereoisomers (d1 / d2: 70 / 30 ratio measured by ^{1}H-NMR on the crude mixture).

^{1}H-NMR (D_2O, 200 MHz) δ (ppm) = 8.36 (s, 0.34H, d1), 8.11 (s, 0.17H, d2), 5.37 (d, *J* = 3.2 Hz, 0.4H, d2), 5.18 (d, *J* = 8.2 Hz, 1H, d1), 5.01-4.78 (m, 2.8H, d1+d2), 4.55 (d, *J* = 7.8 Hz,

0.4H, d2), 4.47 (dd, J = 2.2 Hz and J = 7.8 Hz, 1H, d1), 4.41-4.32 (m, 1.4H, d1+d2), 4.30-4.06 (m, 1.4H, d1+d2), 3.92-3.74 (m, 4.2H, d1+d2), 3.71-3.53 (m, 4.2H, d1+d2), 3.45-3.32 (m, 4.2H, d1+d2), 3.30-3.17 (m, 1.4H, d1+d2); ^{13}C-NMR (D_2O, 50 MHz): **d1**: δ (ppm) = 143.5, 130.3, 101.4, 100.5, 94.1, 75.9, 75.6, 73.4, 73.0, 72.9, 69.5, 67.5, 61.8, 60.7, 56.5; **d2** δ (ppm) = 143.4, 130.2, 101.3, 96.7, 90.8, 76.0, 75.8, 72.4, 71.8, 71.6, 69.8, 68.4, 67.7, 60.6, 60.4; HRMS (ESI): Calcd. for C15H25N3O11Na [M+Na]+:446.1381; Found: 446.1390.

(2*R*,3*R*,4*S*,5*R*,6*R*)-2-(Hydroxymethyl)-6-(4-(hydroxymethyl)-1*H*-1,2,3-triazol-1-yl)tetrahydro-2*H*-pyran-3,4,5-triol (18) [2]**:** A Schlenk tube was charged with an H_2O solution (7 mL) containing the complex **1** (1 mol%,13 mg), azide **8** (410 mg, 2 mmol) and the propargyl alcohol **5** (0.12 mL, 2 mmol), and then capped with a rubber septum. The reaction mixture, protected from light by aluminium foil, was degassed by gentle argon bubbling for 20 minutes, tightly closed with parafilm and then irradiated at 365 nm for 60 min using a TLC lamp placed at ~ 1 cm from the tube. The solvent was evaporated and the residue was taken up with MeOH. Adding ethyl acetate led to the precipitation of the triazole **18** which was recovered by filtration as a white powder (450 mg, 86%).

M.p. 191-193 °C; ^{1}H-NMR (D_2O, 400 MHz) δ (ppm) = 8.24 (s, 1H), 5.71 (d, J = 9.2 Hz, 1H), 4.75 (s, 2H), 4.25 (t, J = 9.6 Hz, 1H), 4.10 (d, J = 3.2 Hz, 1H), 4.01 (t, J = 6.0 Hz, 1H), 3.89 (dd, J = 3.2 Hz and J = 10.0 Hz, 1H284.0849.), 3.79 (d, J = 6.0 Hz, 2H); ^{13}C-NMR (D_2O, 101 MHz) δ (ppm) = 147.2, 123.3, 88.2, 78.5, 73.1, 69.9, 68.7, 61.0, 54.8; HRMS (ESI): Calcd. for $C_9H_{15}N_3O_6Na$ $[M+Na]^+$: 284.0853; Found: 284.0849.

3-(4-(Hydroxymethyl)-1*H*-1,2,3-triazol-1-yl)propan-1-ol (19) [6]**:** A Schlenk tube was charged with a H_2O solution (17 mL) of complex **1** (1 mol %,13 mg), 3-azidopropan-1-ol (505 mg, 5 mmol) and propargyl alcohol **5** (0.28 mL, 5 mmol), and capped with a rubber septum. The reaction mixture, protected from light by aluminum foil, was then degassed by gentle argon bubbling for 20 minutes, tightly closed with parafilm, and then irradiated at 365 nm for

60 min using a TLC lamp placed at ~1 cm from the tube. The desired triazole **19** was obtained as a yellow oil (644 mg, 82%) after purification on silica gel column chromatography (Ethyl acetate / DCM, 50 / 50, v/v).

^{1}H-NMR (CD_3OD, 200 MHz) δ (ppm) = 7.91 (s, 1 H), 4.67 (s, 2H), 4.49 (d, *J* = 7.0 Hz, 2H), 3.55 (t, *J* = 6.0 Hz, 2H), 2.08 (dd, *J* = 12.9 Hz and *J* = 6.5 Hz, 1H); ^{13}C-NMR (CD_3OD, 50 MHz) δ (ppm) = 147.6, 123.0, 57,9, 55.1, 46.8, 32.6; HRMS (ESI): Calcd. for $C_6H_{12}N_3O_2$ $[M+H]^+$: 158.0924; Found: 158.0925.

References

1. Morotti, A. L. M.; Lang, K. L.; Carvalho, I.; Schenkel, E. P.; Bernardes, L. S. C. *Tetrahedron Lett.* **2015,** *56,* 303–307.
2. Harmand, L.; Cadet, S.; Kauffmann, B.; Scarpantonio, L.; Batat, P.; Jonusauskas, G.; McClenaghan, N. D.; Lastécouères, D.; Vincent, J.-M. *Angew. Chem., Int. Ed.* **2012,** *51,* 7137–7141.
3. Ortega-Muñoz, M.; Perez-Balderas, F.; Morales-Sanfrutos, J.; Hernandez-Mateo, F.; Isac-García, J.; Santoyo-Gonzalez, F. *Eur. J. Org. Chem*. 2009, 2454–2473
4. Rauthu, R. S.; Shiao, T. C.; André, S.; Miller, M. C.; Madej, E.; Mayo, K. H.; Gabius, H. J.; Roy, R. *ChemBioChem* **2015,** *16,* 126–139.
5. Shen, H.; Shen, C.; Chen, C.; Wanga, A.; Zhang, P. *Catal. Sci. Technol.,* **2015**, *5*, 2065–2071
6. Girard, C.; Onen, E.; Aufort, M.; Beauvière, S.; Samson, E.; Herscovici, J. *Org. Lett.* **2006,** *8,* 1689–1692.
